# Effects of cat ownership on the gut microbiota of owners

**DOI:** 10.1371/journal.pone.0253133

**Published:** 2021-06-16

**Authors:** Guankui Du, Hairong Huang, Qiwei Zhu, Li Ying

**Affiliations:** 1 Department of Biochemistry and Molecular Biology, Hainan Medical University, Haikou, China; 2 The Key Laboratory of Molecular Biology, Hainan Medical University, Haikou, China; 3 School of Public Health, Hainan Medical University, Haikou, China; 4 Haikou Customs, Haikou, China; Wageningen Universiteit, NETHERLANDS

## Abstract

Pet ownership is an essential environmental exposure that might influence the health of the owner. This study’s primary objectives were to explore the effects of cat ownership on the gut microbial diversity and composition of owners. Raw data from the American Gut Project were obtained from the SRA database. A total of 214 Caucasian individuals (111 female) with cats and 214 individuals (111 female) without cats were used in the following analysis. OTU number showed significant alteration in the Cat group and Female_cat group, compared with that of the no cat (NC) group and Female_ NC group, respectively. Compared with the NC group, the microbial phylum Proteobacteria was significantly decreased in the Cat group. The microbial families *Alcaligenaceae* and *Pasteurellaceae* were significantly reduced, while *Enterobacteriaceae* and *Pseudomonadaceae* were significantly increased in the Cat group. Fifty metabolic pathways were predicted to be significantly changed in the Cat group. Twenty-one and 13 metabolic pathways were predicted to be significantly changed in the female_cat and male_cat groups, respectively. Moreover, the microbial phylum Cyanobacteria was significantly decreased, while the families *Alcaligenaceae*, *Pseudomonadaceae* and *Enterobacteriaceae* were significantly changed in the normal weight cat group. In addition, 41 and 7 metabolic pathways were predicted to be significantly changed in the normal-weight cat and overweight cat groups, respectively. Therefore, this study demonstrated that cat ownership could influence owners’ gut microbiota composition and function, especially in the female group and normal-weight group.

## Introduction

Pet ownership is thought to contribute to human health by reducing stress and improving mental health [1,2]. Experience with cat ownership protects against incident frailty in elderly individuals [3]. Domestic cats may reduce the risk of cardiovascular disease [4]. Sixty percent of cat owners sleep with their cats, which may enhance their sense of security and improve their quality of sleep [5]. However, studies have shown that cat ownership is associated with schizophrenia and allergic diseases [6,7].

Hundreds of millions of microorganisms live in the human intestines [8]. Microbes interact with each other by producing metabolites and forming complex communities [9]. Intestinal microorganisms can provide the host with necessary nutrients, such as vitamin K, B2, B6, and B12 [10]. Intestinal microorganisms can also help the host metabolize and convert various substances, such as polysaccharides, proteins, fats, and vitamins [11]. However, some secondary metabolites produced by microbes are toxic to the host, such as neurotoxin β-methylamino-L-alanine and cardiotoxin trimethylamine N-oxide [12]. Therefore, a well-balanced intestinal microecological environment is critically important to host health [13]. The gut microbiota is susceptible to diverse factors, such as diet, genetics, and exercise [14].

Previous research has shown that pets and their owners may share a common gut microbiota [15]. Exposure to pets can affect the richness and diversity of microbes in the intestines of infants and young children [16,17]. Pets rapidly introduce new microbial taxa into a house [18]. It is speculated that close physical contact with pets, such as hugging, may affect gut microbiota [19]. Therefore, we analyzed the gut microbiota of individuals with cats and compared the results with those for individuals without cats. This study attempted to determine the influence of cat ownership on gut microbial diversity and composition in different groups of individuals and to identify the bacterial phyla and families that were significantly affected.

## Material and method

### Data sources

The American Gut Project (AGP) carried out a questionnaire survey while collecting stool samples. The questionnaire included necessary information such as age, sex, height, and weight and information on lifestyle, dietary habits, and basic diseases. The collection and storage of samples, bacterial DNA extraction, sequencing, and quality assessment were conducted following the standards of the Earth Microbiome Project [20]. The AGP’s original sequencing data were stored in the SRA database (https://www.ncbi.nlm.nih.gov/sra/) and ENA database (https://www.ebi.ac.uk/ena/browser/home) under accession number PRJEB11419. Data from 25,376 individuals were collected by the AGP. However, some data could not be included in this study, including data collected from nonfecal samples, incomplete questionnaires (lack of essential information such as sex, age, and body mass index), patients suffering from serious diseases (such as cancer), patients receiving antibiotic treatment within six months, patients traveling within three months (the changes in the diet might affect the gut microbiota), and samples with low sequencing quality (the total sequencing depth was less than 8000). Finally, we obtained data for a total of 3795 individuals for the following analyses. Through self-reporting, we found 214 individuals who claimed that they owned cats but no other pet (Cat group), while 214 individuals who did not own a pet were matched with the Cat group by gender, body mass index (BMI), and age (no cat (NC) group) (S1 Table).

### Group

The individuals (Table 1) were divided into groups by sex, age, and BMI. Both the cat and NC groups included 111 female individuals and 103 male individuals, 82 overweight individuals (OW), and 132 normal-weight individuals (NW).

**Table 1:** Demographic and anthropometric characteristics of the individuals with or without cat.

	Cat	No Cat	Chi-square	P-Value
Total Number	214	214		
Age	45.1±7.5	46.1±15.4		
Adult_18-60_ (Number)	171	171		
Elderly (Number)	43	43		
BMI	24.8 ±4.4	24.3 ±1.8		
Normal weight (Number)	132	132		
Over weight (Number)	82	82		
Gender				
female (Number)	111	111		
male (Number)	103	103		
Caucasian (Number)	214	214		
Country_residence				
United Kingdom (Number)	90	92	0.015421	0.901172
United States (Number)	124	122	0.010326	0.919063
Diet_type				
Omnivore	169	175	0.058023	0.809649
Vegan & Vegetarian	16	10	1.305548	0.253202
Omnivore but do not eat red meat	15	16	0.030080	0.862310
Vegetarian but eat seafood	14	10	0.631318	0.426872
Not provided	0	3	2.979263	0.084338

### Converting SRA to FASTQ format

To convert SRA data to the FASTQ format, we performed the "fastq-dump.exe" command with the sratoolkit tool (https://trace.ncbi.nlm.nih.gov/Traces/sra/sra.cgi?view=software).

### Data processing

To analyze the 16S rRNA sequence data, we employed QIIME2 software [21]. First, the FASTQ file obtained in the previous step was packaged into a file named demux.qza. Next, the Deblur plug-in was used to perform quality control analysis, and a feature table was established. The "qiime phylogeny align-to-tree-mafft-fasttree" plug-in was used to produce unrooted trees. The "qiime diversity alpha rarefaction" plug-in was used to determine the alpha diversity. The alpha rarefaction visualization operations were applied to analyze qzv files generated in this step through the QIIME tools view command, which provided two α-diversity analyses (operational taxonomy units (OTUs) and Shannon index).

Next, the ‘q2-feature-classifier plug-in’ with the Greengenes 13.8 Database was used to assign taxonomy to the sequences and then map sequences based on taxonomy. To simplify the subsequent analysis, we conducted a screening and retained the taxa found in at least 1% of the samples.

PICRUSt2 was used to predict metagenomic function based on normalized OTU tables [22].

### Statistical analysis

Statistical Analysis of Metagenomic Profiles (STAMP 2.1.3) was used to estimate statistical significance for the relative microbial abundance [23]. The Benjamini-Hochberg false discovery rate (FDR) method was used to calculate adjusted P-values. P<0.05 indicated a significant difference.

## Results

The present study was carried out to characterize cat ownership-induced changes in the properties of the owners’ gut microbiota. As shown in Table 1, age, BMI, sex, ethnicity, country residence, and diet type were not significantly different between the cat and without cat groups.

### The effect of cat ownership on gut microbial composition and function

**Fig 1:**
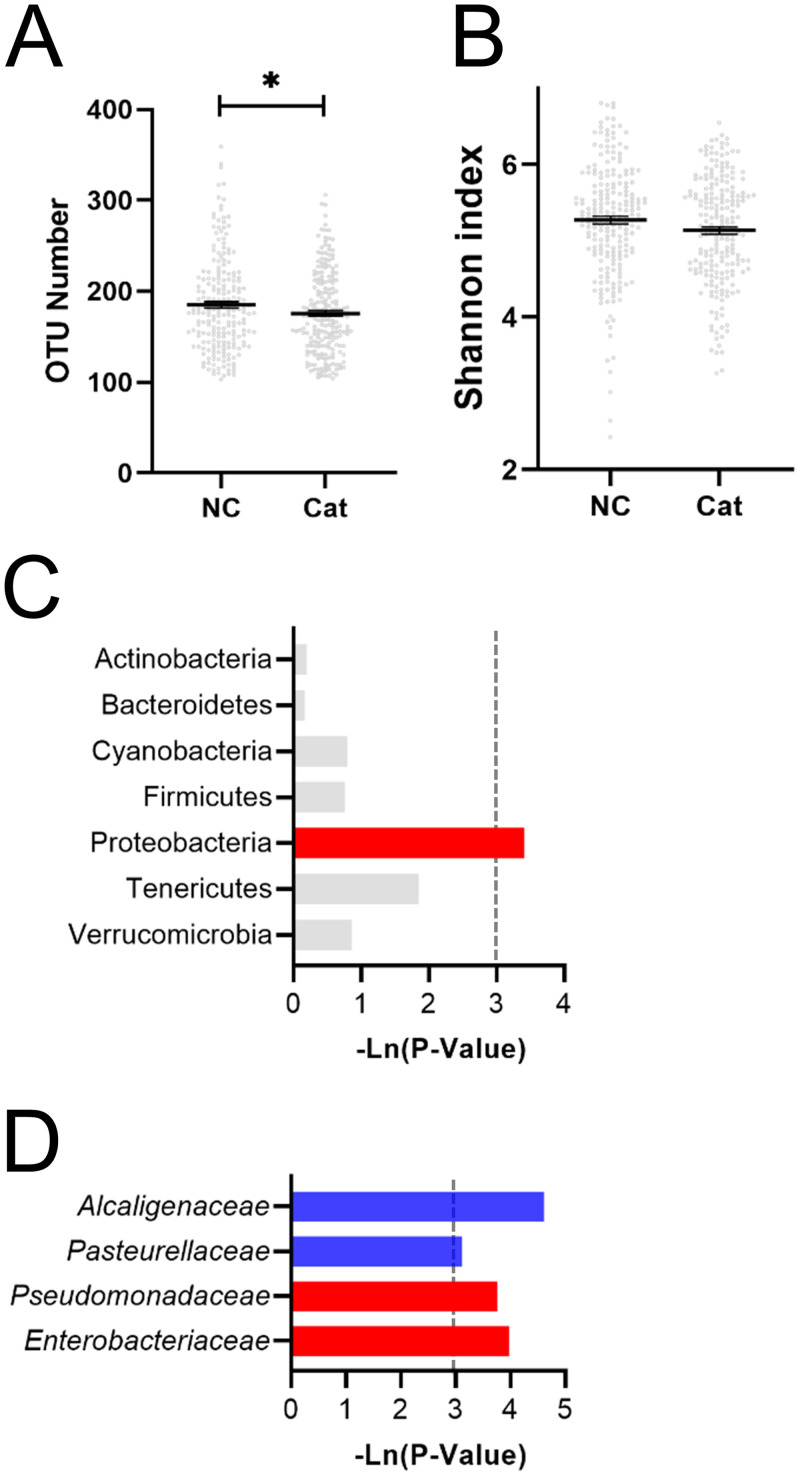
The effect of cat ownership on the composition of the microbiota of all individuals. Cat ownership did not affect the (A) number of OTUs or the (B) Shannon index. The effect of cat ownership on bacteria at the (C) phylum level and (D) family level. The red bar represents a significant increase, while the blue bar represents a significant decrease.

The α-diversity analysis, which could reflect the abundance and diversity of the microbial community, showed that the OTU number was significantly decreased, while the Shannon index (Shannon value was positively correlated with community diversity) was not significantly altered in the cat group compared with the NC group (Fig 1A and 1B). As shown in Fig 1C and 1D, the microbial composition was impacted by owning a cat. At the phylum level, Proteobacteria were significantly induced by cat ownership (Fig 1C). At the family level, the relative abundances of *Alcaligenaceae* and *Pasteurellaceae* were significantly reduced, while those of *Enterobacteriaceae* and *Pseudomonadaceae* were significantly increased (Fig 1D).

**Fig 2:**
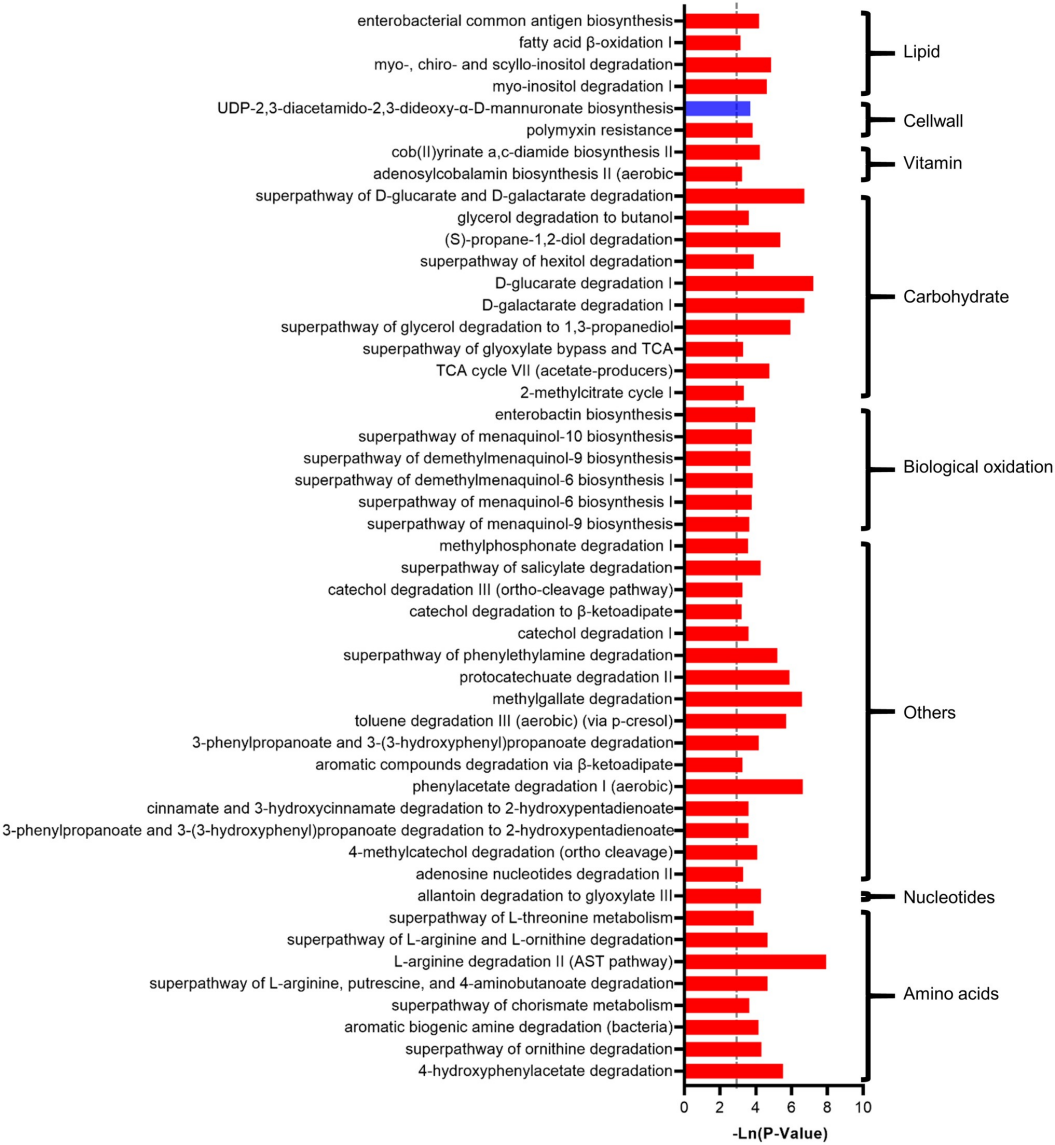
The effect of cat ownership on the function of the microbiota of all individuals. The significant effect of cat ownership on microbial metabolism pathways.

In addition, 50 metabolic pathways were predicted to be significantly changed (P<0.05), which showed increased metabolism of amino acids, nucleotides, biological oxidation carbohydrates, vitamins and lipids (Fig 2). The degradation of L-arginine, L-ornithine, and L-threonine was significantly increased. The degradation of galactarate and glucarate was significantly increased, and the tricarboxylic acid cycle was significantly increased. In addition, the pathway of vitamin B12 biosynthesis was increased, and myo-, chiro- and scyllo-inositol degradation and the fatty acid β-oxidation pathway were increased.

### The effect of cat ownership on the gut microbiota of females and males

**Fig 3:**
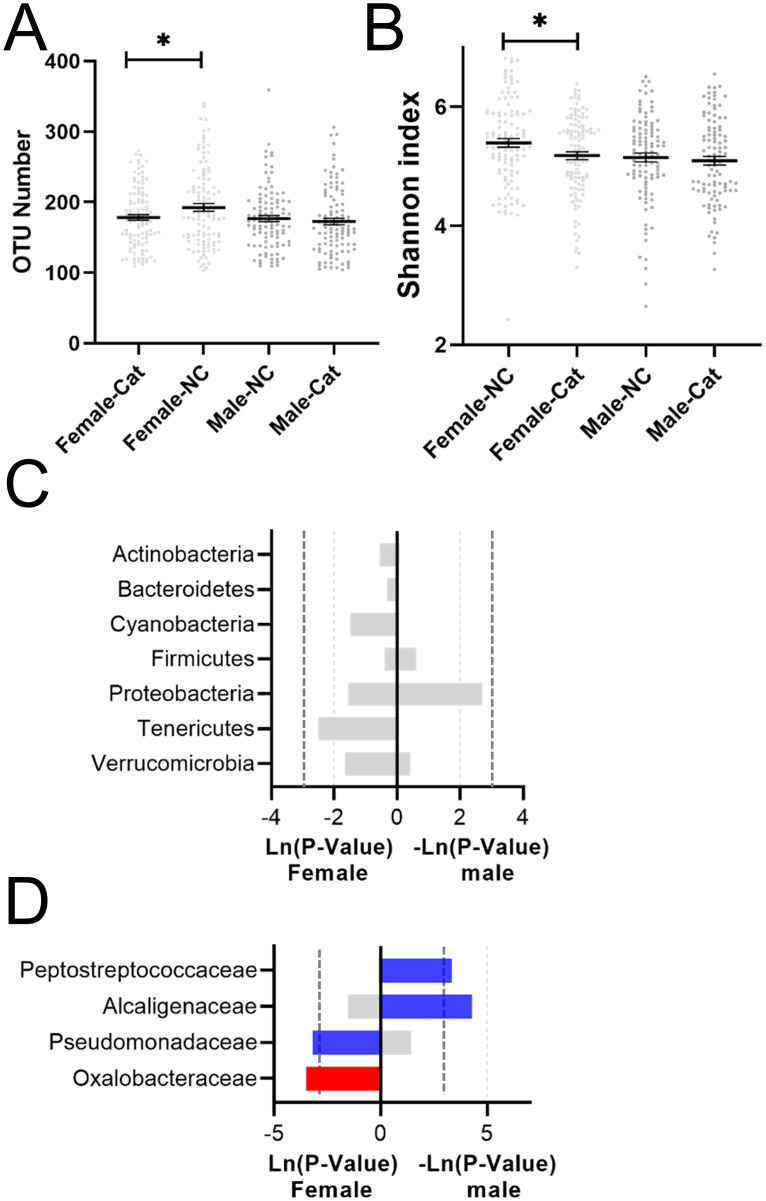
The effect of cat ownership on the composition of the microbiota of female and male individuals. Cat ownership did not affect the (A) number of OTUs or the (B) Shannon index. The effect of cat ownership on bacteria at the (C) phylum level and (D) family level.

The α-diversity analysis showed that the OTU number and the Shannon index were significantly altered in the female_Cat group (Fig 3A and 3B). However, at the phylum level, almost no microbes were significantly changed in the female_Cat and Male_Cat groups (Fig 3C). At the family level, the relative abundance of *Oxalobacteraceae* was significantly increased, while *Pseudomonadaceae* was significantly decreased in the female_cat group compared with the female_NC group. *Alcaligenaceae* and *Peptostreptococcaceae* were significantly decreased in the Male_Cat group compared with the Male_NC group (Fig 3D).

**Fig 4:**
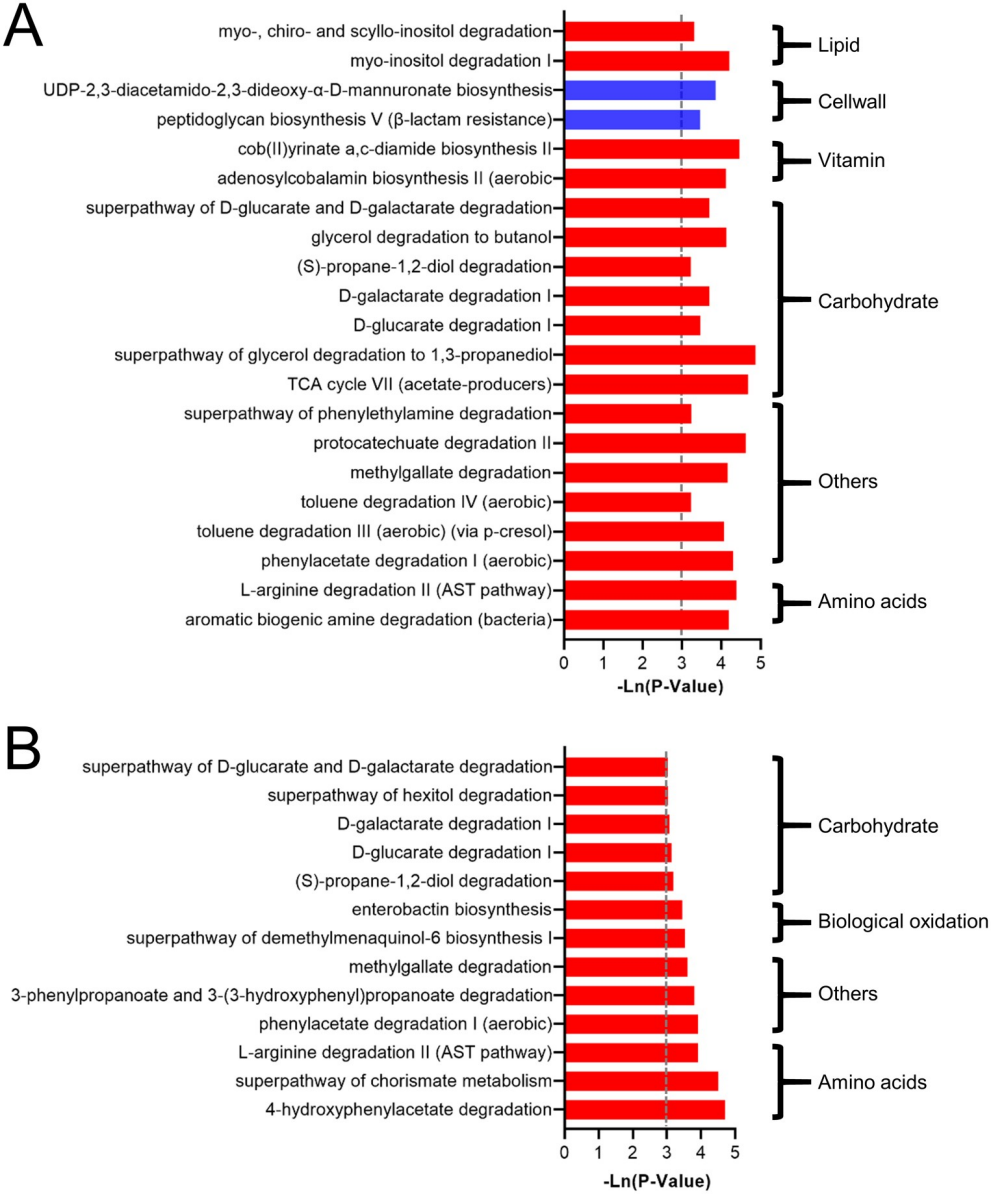
The effect of cat ownership on the composition of the microbiota of female and male individuals. The significant effect of cat ownership on microbial metabolism pathways in (A) female and (B) male individuals.

In addition, 21 and 13 metabolic pathways were predicted to be significantly changed in the female_cat and male_cat groups, respectively (P<0.05) (Fig 4). In the female_cat group, the metabolism of amino acids, carbohydrates, vitamins and lipids was significantly increased. In the male_cat group, the metabolism of amino acids, biological oxidation and carbohydrates were significantly increased.

### The effect of cat ownership on the gut microbiota of the NW and OW groups

**Fig 5:**
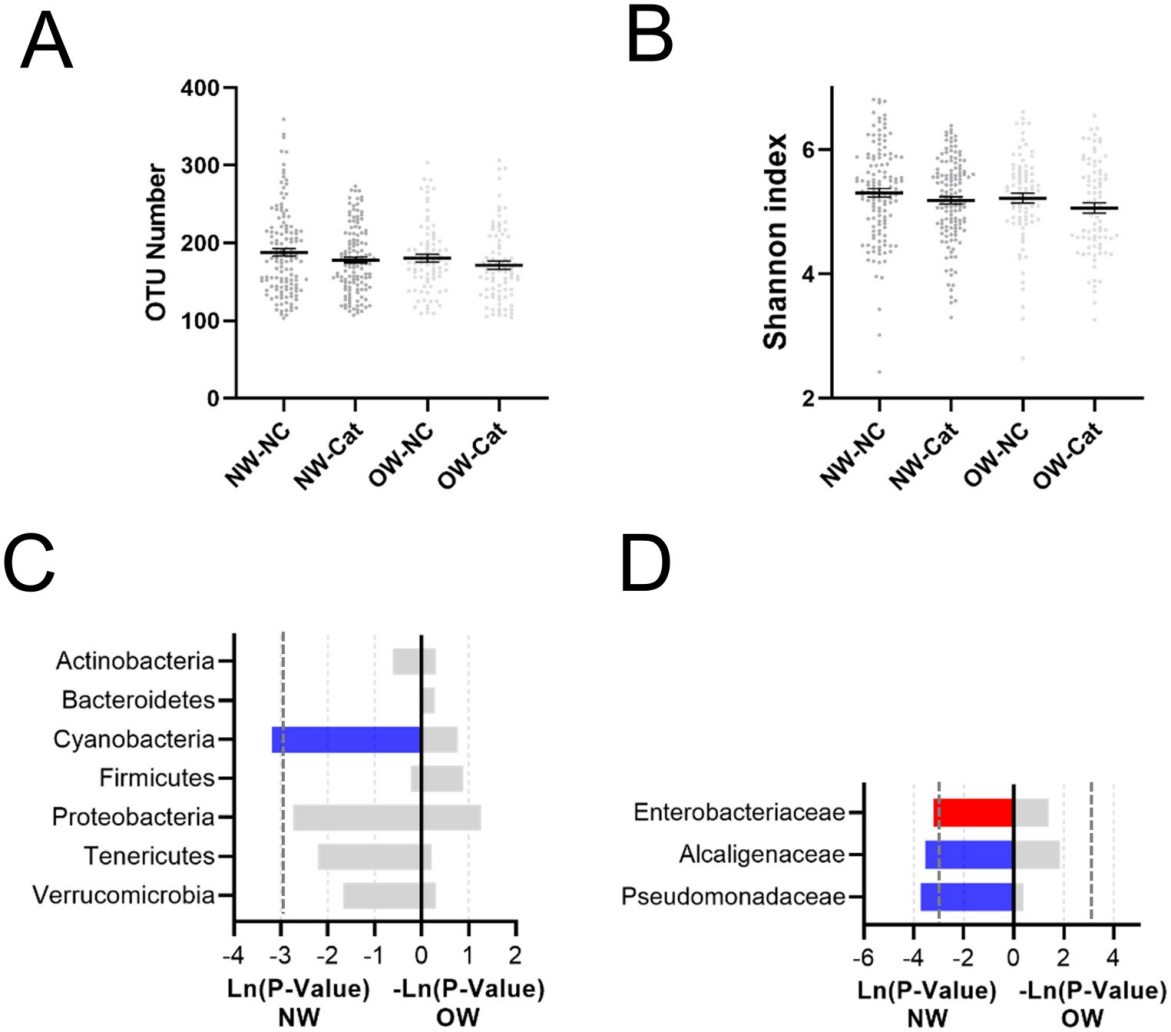
The effect of cat ownership on the microbiota composition of normal-weight and overweight individuals. Cat ownership did not affect the (A) number of OTUs or the (B) Shannon index. The effect of cat ownership on bacteria at the (C) phylum level and (D) family level.

The α-diversity analysis showed that the OTU number and the Shannon index were not significantly altered in the NW and OW groups (Fig 5A and 5B). Moreover, at the phylum level, the relative abundance of Cyanobacteria was significantly decreased in the NW_cat group (Fig 5C). At the family level, the relative abundance of *Enterobacteriaceae* was significantly increased, while that of *Alcaligenaceae* and *Pseudomonadaceae* were significantly decreased in the female_cat group compared with the female_NC group (Fig 5D).

**Fig 6:**
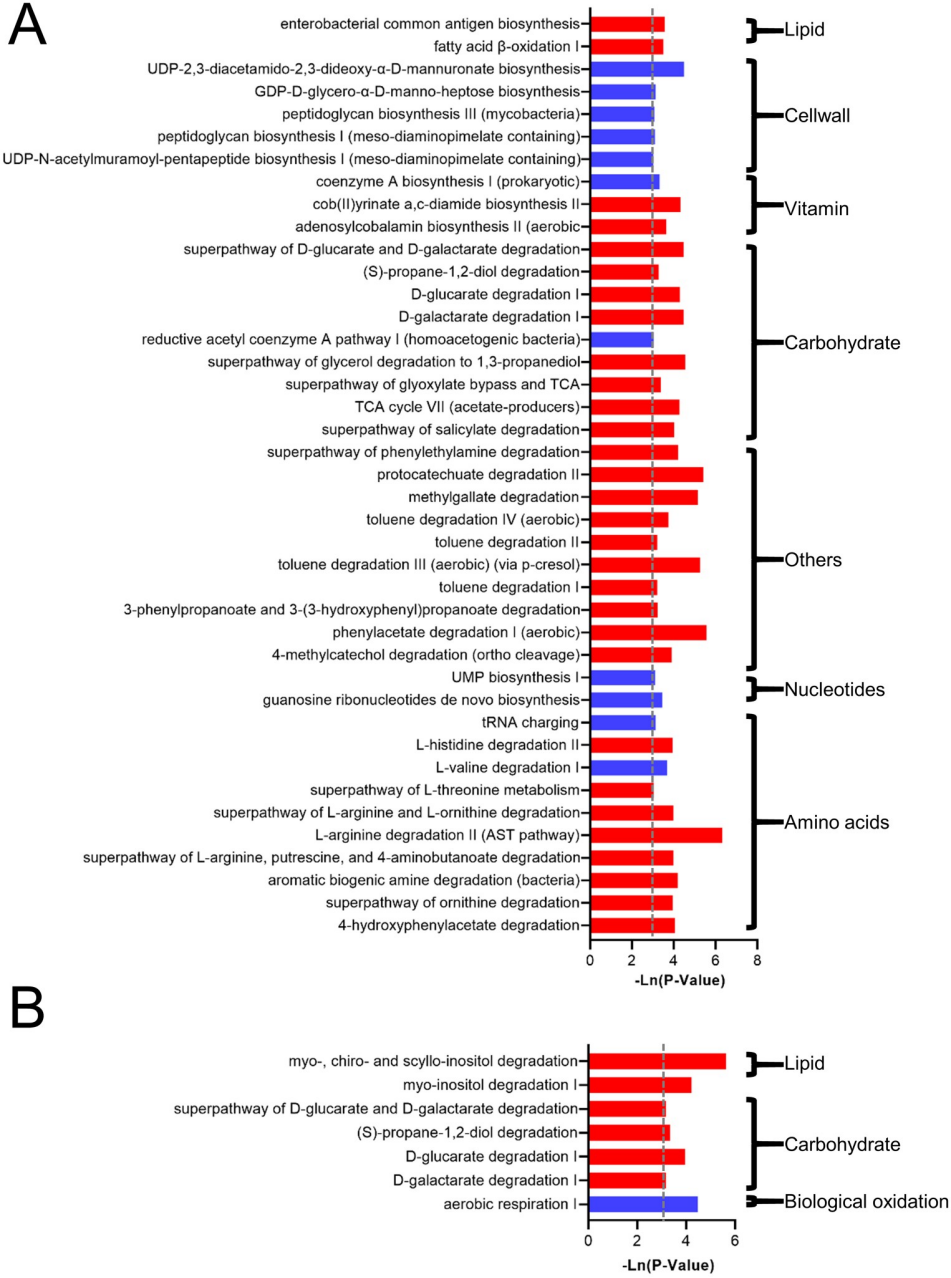
The effect of cat ownership on the microbiota’s function of normal weight and overweight individuals. The significant effect of cat ownership on microbial metabolism pathways, (A) normal-weight and (B) overweight individuals.

In addition, 41 and 7 metabolic pathways were predicted to be significantly changed in the NW_cat and OW_cat groups, respectively (P<0.05) (Fig 6). In the NW_cat group, the metabolism of carbohydrates and lipids was significantly increased, while the metabolism of cell walls, amino acids and nucleotides was significantly decreased. In the OW_cat group, the metabolism of carbohydrates and lipids was significantly increased.

## Discussion

The gut microbiota is affected by diet, drugs, antibiotics, and environmental exposure [24]. One of the environmental exposures that needs attention is pet ownership. This study showed that cat ownership significantly affects the gut microbiota, especially in female and NW individuals.

The present study showed that cat ownership affects not only microbial α-diversity but also the abundance of Proteobacteria, *Alcaligenaceae*, *Pasteurellaceae*, *Enterobacteriaceae* and *Pseudomonadaceae*. A previous study showed that contact with pets can affect a baby’s gut microbiota [25]. Moreover, Proteobacteria includes many pathogenic bacteria, such as *Escherichia coli*, *Salmonella*, *Vibrio cholerae* and *Helicobacter pylori* [26]. Increased *Alcaligenaceae* is associated with diseases such as hyperuricemia and constipation [27]. Increased *Pasteurellaceae* is involved in granulomatosis with vasculitis [28]. Increased *Enterobacteriaceae* is involved in gastritis, schizophrenia, alcoholic hepatitis, and Crohn’s disease [29–32]. Increased *Pseudomonadaceae* is associated with cholangiocarcinoma, end-stage renal disease and Crohn’s disease [32–34]. Therefore, cat ownership may be involved in mediating the abundance of disease-related gut microbes.

Furthermore, the present study revealed that cat ownership had a significant impact on females’ gut microbiota. The α-diverstiy were significantly affected, while more metabolic pathways were predicted to be significantly changed in the female_cat group than in the male_cat group. In Female_OW group, the OTU number and Shannon index was significantly decreased in the Cat group compared with the NC group. However, for overweight women, there were only 11 samples (S2 Table). Moreover, Female_OW and Female_NW accounted for 62.41% and 28.01% in AGP, respectively. In line with the ratio of normal weight to overweight, a cohort of female (19 normal weight female and 11 overweight female) were constructed. The α-diversity analysis showed that the OTU number and the Shannon index were significantly decreased in the female_Cat group (S3 Table). It was reported that females are more likely to own cats [35]. In particular, for females who live alone, having a cat helps reduce loneliness [36,37]. Females are more willing to communicate with cats than men [38]. Therefore, the greater exposure of females to cats means that the gut microbiota is relatively more susceptible to being affected.

The present study showed that the phylum Cyanobacteria was significantly reduced, while the families *Enterobacteriaceae*, *Alcaligenaceae* and *Pseudomadaceae* were significantly affected in the NW_cat group. Moreover, 41 metabolic pathways were predicted to be significantly changed in the NW_cat group, which was far more than that in the OW_cat group. However, there were only 32 samples in male_NW (S4 Table). We constructed a cohort of normal weight with 32 female individuals and male individuals. As shown in S5 Table, the α-diversity analysis showed that the OTU number was significantly decreased in the NW_Cat group. Moreover, at the phylum level, the relative abundance of Cyanobacteria was significantly decreased in the NW_cat group. It has been reported that the relative ratio of Bacteroidetes decreased in obese individuals [39]. However, a recent study showed no association between pet ownership and obesity [40]. Therefore, from the perspective of gut microbiota, our study not only supports that there might be no correlation between cat ownership and obesity but also clarifies that cat ownership can affect the structure and function of gut microbiota in NW individuals.

Functional predictions indicated that cat ownership would lead to increased synthesis of B vitamins, amino acids and carbohydrate metabolism. Moreover, SCFA-related pathways (4-hydroxyphenylacetate degradation, TCA cycle VII (acetate producers), and glycerol degradation to butanol) were significantly increased. As the biosynthetic precursor of cofactors, vitamins play a vital role in organisms. The gut microbiota can provide various vitamins for the host [41]. Increasing glucose metabolism in the gut microbiota may be beneficial to host blood glucose control [42]. SCFAs can participate in intestinal epithelial energy supply, affect the intestinal environment (such as pH and electrolyte balance), and regulate host material and energy metabolism. SCFAs are related to the occurrence of various energy metabolism diseases. SCFAs have anti-inflammatory effects. Therefore, the influence of cat ownership on gut microbiota function may affect the health of the owner.

Recent studies have been undertaken to focus on the gut microbiota of cats. The main microbial phyla in cats were Firmicutes, Bacteroidetes, Proteobacteria, Fusobacteria, and Actinobacteria [43]. The prominent microbial families in cats were *Prevotellaceae*, *Peptostreptococcaceae*, *Veillonellaceae*, *Lachnospiraceae*, *Clostridiales*, *and Erysipelotrichaceae* [44]. Microorganisms can spread through the air and touch, making it possible to exchange microorganisms between humans and animals [45,46]. This study showed that Firmicutes, Bacteroidetes, Proteobacteria, Tenericutes, and Verrucomicrobia were the 5 most abundant microbial phyla, while *Ruminococcaceae*, *Bacteroidaceae*, *Lachnospiraceae*, *Prevotellaceae*, and *Enterobacteriaceae* were the dominant microbial families. Thus, the gut microbiota of cats and humans are quite different. Therefore, ownership of cats affects the human gut microbiota in multiple ways, such as contact with the flora on the cat’s hair and the impact of pet companionship on the spirit, worthy of further study.

It has been reported that the gut microbiota is affected by various factors, such as diet, race, and antibiotic history [47,48]. Therefore, this study included only Caucasian individuals who live in the United States and the United Kingdom. In addition, the diet of individuals with cats and without cats was not significantly different (Table 1 and S6 Table). However, in the AGP questionnaire, intimacy with cats, the manner of living with cats, and the average time to get along with cats were not involved, which may impact the results. Ultimately, this study found that cat ownership plays a role in modulating owners’ gut microbiota, which is important for further studies.

## Conclusion

In general, cat ownership is a factor that needs to be considered and can affect microbial diversity and composition. The ownership of cats has a significant influence on the gut microbiota of females and NW individuals, but it is not closely related to the gut microbiota of OW individuals. In addition, multiple microbial metabolic pathways were affected by cat ownership. In future studies, a larger-scale and more detailed investigation can verify the impact of cat ownership on owners’ gut microbiota.

## Supporting information

S1 Table Basic information, OTU number, shonnon index and OTU taxonomy of 428 samples. (XLSX)

S2 Table Demographic and anthropometric characteristics of the female with or without cat. (DOCX)

S3 Table Effects of cat ownership on gut microbiota in subgroups. (DOCX)

S4 Table Effects of cat ownership on gut microbiota in female. (DOCX)

S5 Table Effects of cat ownership on gut microbiota in normal weight. (DOCX)

S6 Table Demographic and anthropometric characteristics of the normal weight with or without cat. (DOCX)
