# Supplementary material for: Effects of cat ownership on the gut microbiota of owners
Source: PLoS One. 2021 Jun 16;16(6):e0253133. doi: 10.1371/journal.pone.0253133 (PMC8208556; doi:10.1371/journal.pone.0253133)
Supplement: S2 Table — (DOCX) [file pone.0253133.s002.docx]

**Table S2. Demographic and anthropometric characteristics of the female with or without cat.**

|  | Cat | No Cat | Chi-square | P-Value |
| --- | --- | --- | --- | --- |
| Female | 111 | 111 |  |  |
| Age | 44.1±14.0 | 44. 7±15.3 |  |  |
| Adult_18-60_ (Number) | 95 | 95 |  |  |
| Elderly (Number) | 16 | 16 |  |  |
| BMI | 22.2 ±2.4 | 22.2 ±1.8 |  |  |
| Normal weight (Number) | 100 | 100 |  |  |
| Over weight (Number) | 11 | 11 |  |  |
| Caucasian (Number) | 111 | 111 |  |  |
| Country_residence |  |  |  |  |
| United Kingdom (Number) | 50 | 47 | 0.064576 | 0.799404 |
| United States (Number) | 61 | 64 | 0.046067 | 0.830054 |
| Diet_type |  |  |  |  |
| Omnivore | 82 | 87 | 0.084004 | 0.771943 |
| Vegan & Vegetarian | 9 | 6 | 0.562115 | 0.453409 |
| Omnivore but do not eat red meat | 10 | 7 | 0.491832 | 0.483111 |
| Vegetarian but eat seafood | 10 | 9 | 0.048483 | 0.825724 |
| Not provided | 0 | 2 | 1.982301 | 0.159148 |
