# Supplementary material for: Effects of cat ownership on the gut microbiota of owners
Source: PLoS One. 2021 Jun 16;16(6):e0253133. doi: 10.1371/journal.pone.0253133 (PMC8208556; doi:10.1371/journal.pone.0253133)
Supplement: S3 Table — (DOCX) [file pone.0253133.s003.docx]

**Table S3 Effects of cat ownership on gut microbiota in subgroups**

|  | **Female_NW** | |  | **Female_OW** | |  | **Male_NW** | |  | **Male_OW** | |
| --- | --- | --- | --- | --- | --- | --- | --- | --- | --- | --- | --- |
|  | **NC** | **Cat** |  | **NC** | **Cat** |  | **NC** | **Cat** |  | **NC** | **Cat** |
| Number | 100 | 100 |  | 11 | 11 |  | 32 | 32 |  | 71 | 71 |
| OTU | 190.2±56.2 | 181.4±42.7 |  | 215.3±57.1 | 151.8±28.7** |  | 180.8±52.7 | 167.9±41.5 |  | 175.0±40.4 | 174.5±50.4 |
| Shannon index | 5.374±0.749 | 5.215±0.681 |  | 5.570±0.687 | 4.860±0.680* |  | 5.101±0.811 | 5.091±0.641 |  | 5.169±0.719 | 5.095±0.764 |
| Phylum (P<0.05) | N | |  | Tenericutes↓ | |  | N | |  | N | |
| Family (P<0.05) | Tissierellaceae↓ Pseudomonadaceae↑ | |  | Barnesiellaceae↓ Oxalobacteraceae↓ Micrococcaceae↓ | |  | Alcaligenaceae↓ | |  | N | |

*P < 0.05 and ** P < 0.01 for Cat group compared with NC group. ↓ indicates a significant decrease in the abundance in Cat group compared with NC group.↑ indicates a significant increase in the abundance in Cat group compared with NC group.
