# Supplementary material for: Effects of cat ownership on the gut microbiota of owners
Source: PLoS One. 2021 Jun 16;16(6):e0253133. doi: 10.1371/journal.pone.0253133 (PMC8208556; doi:10.1371/journal.pone.0253133)
Supplement: S4 Table — (DOCX) [file pone.0253133.s004.docx]

**Table S4 Effects of cat ownership on gut microbiota in female**

|  | **Female** | |  |
| --- | --- | --- | --- |
|  | **NC** | **Cat** |  |
| Number | 30 | 30 |  |
| OTU | 201.5±60.3 | 169.5±43.6* |  |
| Shannon index | 5.458±0.725 | 5.068±0.759* |  |
| Phylum (P<0.05) | N | |  |
| Family (P<0.05) | Fusobacteriaceae↑  S24-7↓  Porphyromonadaceae↑  Oxalobacteraceae↓ | |  |

*P < 0.05 for Cat group compared with NC group. ↓ indicates a significant decrease in the abundance in Cat group compared with NC group.↑ indicates a significant increase in the abundance in Cat group compared with NC group.
