# Supplementary material for: Effects of cat ownership on the gut microbiota of owners
Source: PLoS One. 2021 Jun 16;16(6):e0253133. doi: 10.1371/journal.pone.0253133 (PMC8208556; doi:10.1371/journal.pone.0253133)
Supplement: S5 Table — (DOCX) [file pone.0253133.s005.docx]

**Table S5 Effects of cat ownership on gut microbiota in normal weight**

|  | **Normal weight** | |  |
| --- | --- | --- | --- |
|  | **NC** | **Cat** |  |
| Number | 64 | 64 |  |
| OTU | 187.9±56.1 | 170.2±43.3* |  |
| Shannon index | 5.223±0.765 | 5.041±0.739 |  |
| Phylum (P<0.05) | Cyanobacteria↓ | |  |
| Family (P<0.05) | Clostridiaceae↓ | |  |

*P < 0.05 for Cat group compared with NC group. ↓ indicates a significant decrease in the abundance in Cat group compared with NC group.
