# Supplementary material for: Effects of cat ownership on the gut microbiota of owners
Source: PLoS One. 2021 Jun 16;16(6):e0253133. doi: 10.1371/journal.pone.0253133 (PMC8208556; doi:10.1371/journal.pone.0253133)
Supplement: S6 Table — (DOCX) [file pone.0253133.s006.docx]

**Table S6. Demographic and anthropometric characteristics of the normal weight with or without cat.**

|  | Cat | No Cat | Chi-square | P-Value |
| --- | --- | --- | --- | --- |
| Normal weight | 132 | 132 |  |  |
| Gender |  |  |  |  |
| Female(Number) | 100 | 100 |  |  |
| Male(Number) | 32 | 32 |  |  |
| Age | 46.5±14.2 | 46. 4±16.1 |  |  |
| Adult_18-60_ (Number) | 105 | 105 |  |  |
| Elderly (Number) | 27 | 27 |  |  |
| BMI | 22.0 ±1.8 | 22.2 ±1.6 |  |  |
| Caucasian (Number) | 132 | 132 |  |  |
| Country_residence |  |  |  |  |
| United Kingdom (Number) | 56 | 65 | 0.459283 | 0.497959 |
| United States (Number) | 76 | 67 | 0.367596 | 0.544318 |
| Diet_type |  |  |  |  |
| Omnivore | 96 | 103 | 0.140432 | 0.707852 |
| Vegan & Vegetarian | 11 | 8 | 0.441932 | 0.506192 |
| Omnivore but do not eat red meat | 12 | 11 | 0.039994 | 0.841491 |
| Vegetarian but eat seafood | 13 | 8 | 1.103096 | 0.293588 |
| Not provided | 0 | 2 | 1.985075 | 0.158857 |
